# T Lymphocytes Promote the Antiviral and Inflammatory Responses of Airway Epithelial Cells

**DOI:** 10.1371/journal.pone.0026293

**Published:** 2011-10-14

**Authors:** Lan Jornot, Samuel Cordey, Assunta Caruso, Christine Gerber, Marija Vukicevic, Caroline Tapparel, Laurent Kaiser, Danielle Burger, Eddy Roosnek, Jean Silvain Lacroix, Thierry Rochat

**Affiliations:** 1 Division of Pulmonary Medicine, Geneva University Hospitals, Geneva, Switzerland; 2 Laboratory of Virology, Division of Infectious Diseases, Geneva University Hospitals, Geneva, Switzerland; 3 Division of Hematology, Geneva University Hospitals, Geneva, Switzerland; 4 Division of Immunology and Allergy, Geneva University Hospitals, Geneva, Switzerland; 5 Rhinology-Olfactology Unit, Geneva University Hospitals, Geneva, Switzerland; National Jewish Health, United States of America

## Abstract

**Hypothesis:**

T cells modulate the antiviral and inflammatory responses of airway epithelial cells to human rhinoviruses (HRV).

**Methods:**

Differentiated primary human nasal epithelial cells (HNEC) grown on collagen-coated filters were exposed apically to HRV14 for 6 h, washed thoroughly and co-cultured with anti-CD3/CD28 activated T cells added in the basolateral compartment for 40 h.

**Results:**

HRV14 did not induce IFNγ, NOS2, CXCL8 and IL-6 in HNEC, but enhanced expression of the T cell attractant CXCL10. On the other hand, HNEC co-cultured with activated T cells produced CXCL10 at a level several orders of magnitude higher than that induced by HRV14. Albeit to a much lower degree, activated T cells also induced CXCL8, IL-6 and NOS2. Anti-IFNγ antibodies and TNF soluble receptor completely blocked CXCL10 upregulation. Furthermore, a significant correlation was observed between epithelial CXCL10 mRNA expression and the amounts of IFNγ and TNF secreted by T cells. Likewise, increasing numbers of T cells to a constant number of HNEC in co-cultures resulted in increasing epithelial CXCL10 production, attaining a plateau at high IFNγ and TNF levels. Hence, HNEC activation by T cells is induced mainly by IFNγ and/or TNF. Activated T cells also markedly inhibited viral replication in HNEC, partially through activation of the nitric oxide pathway.

**Conclusion:**

Cross-talk between T cells and HNEC results in activation of the latter and increases their contribution to airway inflammation and virus clearance.

## Introduction

Common colds caused by viral respiratory infections are the most frequent acute respiratory illness in humans. They can be caused by a variety of different viruses including coronaviruses, parainfluenza virus and respiratory syncytial viruses but infection with rhinoviruses (HRV) of the Picornaviridae family is predominant [1]. The antiviral response depends on the rapid production of interferons (IFNs), which are classified as type 1 IFNs (IFNα/β) and type 2 IFN (IFNγ) based on the receptor complex used for signalling as well as on their sequence homology [2], [3]. Almost every nucleated cell produces IFNα/β when infected by a virus, whereas IFNγ is mainly produced by T cells and NK cells. IFNα/β mount robust host responses against viruses by inducing a wide variety of antiviral proteins including ribonucleases that digest viral double stranded RNA, and the antiviral protein kinase PKR which induces apoptosis. Inducible nitric oxide synthase 2 (NOS2) is also an important component of the innate immune response [4], [5], as it exerts direct antiviral activity by nitrolysating key thiol residues in viral proteases.

Because respiratory epithelial cells are the main target for HRV infection, numerous studies examined the primary direct airway epithelial response to HRV exposure. In vitro infection of airway epithelial cells with rhinovirus induces the production of a wide range of antiviral molecules including INFβ [6], [7], β-defensins [8], [9] as well as NOS2 [10], or proinflammatory chemokines and cytokines that attract and activate cells of the immune system, including CXCL8, CXCL5, CXCL10, CCL5 and IL-6 [11]–[13]. During HRV infections in vivo, levels of epithelial NOS2 induction correlate with levels of exhaled nitric oxide (NO), and subjects with the highest levels of exhaled NO clear the virus more rapidly and have fewer symptoms [14]. CXCL8 and IL-6 are present in nasal secretions and sputum of HRV-infected individuals [15]–[20].

In the present study, we assessed the hypothesis that the antiviral and inflammatory responses of upper airway epithelial cells to human rhinoviruses are modulated by activated T cells. We developed an in vitro model with primary fully differentiated nasal epithelial cells cultured on semi-permeable membranes which separated them from the co-cultured T cells. Our data revealed active cross-talk between epithelial cells and T lymphocytes, which impacted inflammation and virus clearance.

## Materials and Methods

### Epithelial cells culture

Human airway epithelial cells were obtained from patients after partial middle turbinectomies. Patients gave informed verbal consent which was documented in the patient's chart. Both the verbal consent procedure and the present protocol were specifically approved by our institution's ethical committee. Because material provided by surgical samples was handed anonymously to the research laboratory, the requirement for written consent was waived by the ethical review board. The name of the institutional ethical review board is: Commission Centrale d'Ethique de la Recherche, Hôpitaux Universitaires de Genève. Cells were isolated by pronase (Roche, Mannheim, Germany) digestion, and seeded onto collagen-coated Millicell polycarbonate filters (Millipore, MA, USA) as described [21]. Twenty four hours after plating, the mucosal media is removed and the cells are allowed to grow with an air-liquid interface which permits the cells to develop a morphological and functional phenotype that closely resembles in vivo airway epithelium. The culture media consisted of a 1∶1 mix of DMEM:F12 (Life Technologies, CA, USA), 100 U/ml penicillin, 100 µg/ml streptomycin and 50 µg/ml fungizone (DMEM:F12/PSF), 2% Ultroser G (Pall-Biosepra, Cergy-Saint-Christophe, France). Cells were used after 2–3 weeks of culture.

### T cell preparation and activation

Human peripheral blood mononuclear cells (PBMC) were prepared from whole blood of normal individuals using Ficoll Hypaque density centrifugation. T cells were isolated from PBMC by negative immunomagnetic selection using the Dynal T cell negative isolation kit (Life Technologies, CA, USA). T cells thus isolated were activated with CD3/CD28 T Cell Expander artificial antigen-presenting cell beads (Life Technologies, CA, USA) at a ratio of 2∶1.

### T cell clones preparation

T cell clones were generated from PBMC of a normal individual upon antigen activation and cloning by limiting dilution in RPMI 1640 medium supplemented with penicillin (50 U/ml), streptomycin (50 µg/ml), 5% human serum, IL-2 (20 U/ml) and irradiated (3500 rads) autologous PBMC with phytohaemagglutinin (1 µg/ml). Growing cells were further expanded and activated with CD3/CD28 T Cell Expander artificial antigen-presenting cell beads (Life Technologies, CA, USA) at a ratio of 1∶1.

### Viruses

Human rhinovirus type 14 (HRV14) was obtained from the American Type Culture Collection, and propagated by infection of Hela-Ohio cells (kindly provided by Prof. FG Hayden, University of Virginia, Charlottesville, VA, USA) in MEM containing 10% FCS. Cells were infected for 18 h at 33°C, washed and further cultured in McCoy's 5A Medium (Invitrogen, Life Technologies, CA, USA), 2% FCS for 2 days at 33°C. Virus containing supernatants were then collected. The viral stocks were partially purified and concentrated using the Amicon Ultra-4 centrifugal filter devices (Millipore, MA, USA) with a molecular weight cut-off of 100 kDa, according to the manufacturer's instructions. Viruses were titrated on HeLa-Ohio cells to ascertain tissue culture infective dose 50 (TCID_50_), which was calculated using the formula of Reed and Muench [22].

### Treatments of HNEC

All treatments were done in DMEM:F12/PSF without Ultroser G.

#### HRV14 infection

HRV14 in a 100 µl volume was added apically to HNEC at a concentration of 0.5×10^5^ TCID_50_/ml. Cells were incubated with the viruses at 33°C for 6 h. To determine the viral load of the cells, after infection HNEC were washed extensively, and incubated at 33°C for 18 h, 40 h and 60 h in DMEM:F12/PSF with an air liquid interface. HNEC-T cells co-culture experiments were performed at 33°C for 40 h in the absence or presence of T cells.

#### HNEC-T cells co-cultures

For all experiments except the dose-response experiment, 0.5×0^6^ of purified non-activated and CD3/CD28 activated T cells or T cell clones were added in the basolateral compartment. For the dose-response experiment, increasing amounts of non-activated and activated T cells (0.1, 0.5, 2.5 and 5×10^6^ T cells) were used per well. For each experiment, HNEC obtained from biopsies of one anonymous donor were co-cultured with T cells isolated from PBMC of one anonymous healthy donor. Since HNEC were grown on Millicell inserts and T cells were present only in the lower chamber, there was no direct contact between cell populations. In parallel, unstimulated and activated T cells were also cultured separately without HNEC.

#### Incubation with IFNγ neutralizing antibody

HNEC were co-cultured with activated T cells in the absence or presence of either isotype matched IgG or neutralizing anti-IFNγ (1 µg/ml, RD Systems, Abingdon, UK).

#### Incubation with TNF soluble receptor

HNEC were co-cultured with activated T cells in the absence or presence of 10^−7^ M TNF soluble receptor p75 (a kind gift from Amgen, CA, USA).

#### Fluticasone propionate (FP) treatment

Epithelial cells were pretreated apically and basolaterally with 500 nM FP (Sigma-Aldrich, Buchs, Switzerland) for 24 h prior to exposure to HRV14. FP was maintained in the basolateral compartment throughout the 40 h post-infection in the absence or presence of activated T cells.

#### N6-(1-Iminoethyl)-L-lysine hydrochloride (L-NIL) treatment

L-NIL (Calbiochem, Merck Chemicals, Nottingham, UK), a selective inhibitor of inducible nitric oxide synthase, was present (100 µM) during the 6 h HRV infection period, and throughout the 40 h post-infection period in the absence and presence of activated T cells.

#### Treatment with recombinant cytokines

HNEC were exposed basolaterally to IFNγ plus TNF (50 ng/ml each, Sigma-Aldrich, Buchs, Switzerland) for 48 h.

After treatments, apical washings and basolateral media were collected, centrifuged at 1000 rpm at 4°C to remove cell debris and T cells (co-cultures experiments), stored at −20°C for chemokines/cytokines measurement. Epithelial cells were scrapped from the inserts in RNA lysis buffer and stored at −20°C for RNA extraction. For T cells cultures alone, the culture media containing T cells were harvested and centrifuged at 1000 rpm. The supernatants were saved for chemokines/cytokines measurement, and the T cells pellets were lysed in RNA lysis buffer. The anti-CD3/CD28 coated beads were removed from the lysates using the Dynamag-15 magnet (Life Technologies, CA, USA).

### RNA extraction and quantitative real time PCR

Total RNA was extracted from epithelial cells and T lymphocytes using the RNeasy Micro kit (Qiagen, CA, USA) following the manufacturer's instructions, and quantitated with standard UV absorbance measurements. Complementary DNA was synthesized with an oligo(dT) primer and Superscript II, under standard conditions. mRNA levels for CXCL10, CXCL8, IL-6, IFNβ and IFNγ were quantified relative to 18S RNA, using SYBR Green-based quantitative real time PCR on a GeneAmp 5700 Sequence Detection System (Applied Biosystems, Life Technologies, CA, USA). The primers and SYBR Green/Rox PCR master mixes were from SABiosciences (Qiagen, CA, USA).

HRV14 RNA was quantified relative to 18S RNA by real-time PCR using the Panenterhino/Ge/08 combination, as previously described [23].

### Cytokines and chemokines determination

Culture media were assayed for CXCL8, CXCL10, IL-6, and IFNγ proteins using the Ten-Plex Bead Immunoassay and methods provided by the manufacturer (BioSource, Life Technologies, CA, USA). TNF amounts released in the culture media by activated T cells and T cell clones were measured by ELISA (eBioscience, Vienna, Austria). In HNEC/T cells co-cultures experiments, the amounts of cytokines/chemokines secreted by epithelial cells in the basolateral compartments were determined by subtracting the amounts of cytokines/chemokines measured in the culture media of activated T cells cultures alone.

### Nitrite determination

Levels of nitrite, the stable oxidation product of NO, in the apical and basolateral compartments were measured using a colorimetric assay based on the Griess reaction (Molecular Probes, Life Technologies, CA, USA).

### Statistical analysis

The significance of the difference between groups (p<0.05) was determined with the nonparametric Kruskal-Wallis and Mann-Whitney tests for unpaired samples using the Statview program for Windows (Version 5.0.1, SAS Institute Inc.). Data are presented as boxplots with the 25^th^, 50^th^ (median), 75^th^ percentiles, the maximum and the minimum values.

## Results

### HRV14 replicate in differentiated HNEC

To ascertain that differentiated HNEC were infected after a 6 h period incubation with 5000 TCID_50_ HRV14, viral RNA levels were determined in cells by real-time RT-PCR at 18 h, 40 h and 60 h post-infection. There was a time-dependent increase in intracellular viral RNA, by 7.5 fold (median) at 40 h (min 1.4, max 34, n = 8), and 65 fold (median) at 60 h (min 1.5, max 209; n = 8) compared with that observed at 18 h post-infection.

Since several previous in vivo experimental HRV inoculation studies reported that nasal viral titers peaked on day 2 post-inoculation [20], [24], [25] and were correlated with peak symptom scores [20], [24], we chose the 40 h post-infection time point for HNEC-T cells co-cultures experiments.

### Activated T cells, not HRV14 induce epithelial NOS2 mRNA expression and NO release

**Figure 1:**
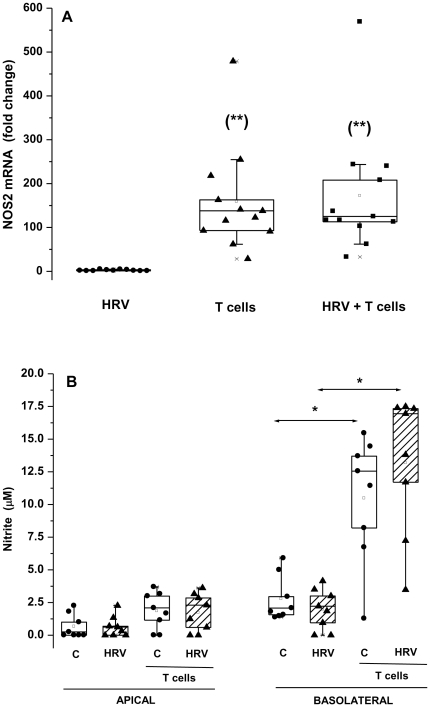
Epithelial NOS2 mRNA expression and NO release are induced by T cells but not HRV14. HNEC were either not infected (C) or exposed apically to HRV14 alone (HRV), or co-cultured with activated T cells alone (T cells), or exposed to HRV14 washed thoroughly then co-cultured with activated T cells (HRV+T cells). (A) mRNA was determined by real-time PCR and data represented as fold increase relative to non-infected cells cultured without T cells. (B) NO secretion is expressed as the amount of nitrite (the stable oxidation product of NO) released in the apical and basolateral compartments (µM). Shown are the boxplots with the 25^th^, 50^th^ (median), 75^th^ percentiles, the mean (open square), the maximum and the minimum values. *p<0.05. **p<0.05 compared with HRV-infected cells.

The antiviral factors IFNβ and NOS2 are important in immediate innate immune response. We assessed their production in HNEC exposed to HRV14, activated T cells, or both. IFNβ mRNA expression was not affected by HRV14, activated T cells or both stimuli (data not shown). A short term exposure to HRV14 did not affect epithelial NOS2 mRNA expression (Fig. 1A) and NO release by HNEC (Fig. 1B). Activated T cells in co-culture with HNEC significantly induced epithelial NOS2 mRNA level (Fig. 1A), corroborated by an increase in the basolateral secretion of nitrite (Fig. 1B). Unstimulated T cells showed no stimulatory effects (data not shown).

### Both HRV14 and activated T cells induce epithelial CXCL10, but only activated T cells induce CXCL8 and IL-6

Chemokines and cytokines that permit recruitment and activation of immune cells are essential to adaptive immune responses to viral infections. CXCL10 and CXCL8 exert chemotactic acitivity towards activated T lymphocytes and neutrophils, respectively. We assessed their production, and that of the pro-inflammatory cytokine IL-6 in HNEC exposed to HRV14, activated T cells, or both.

**Figure 2:**
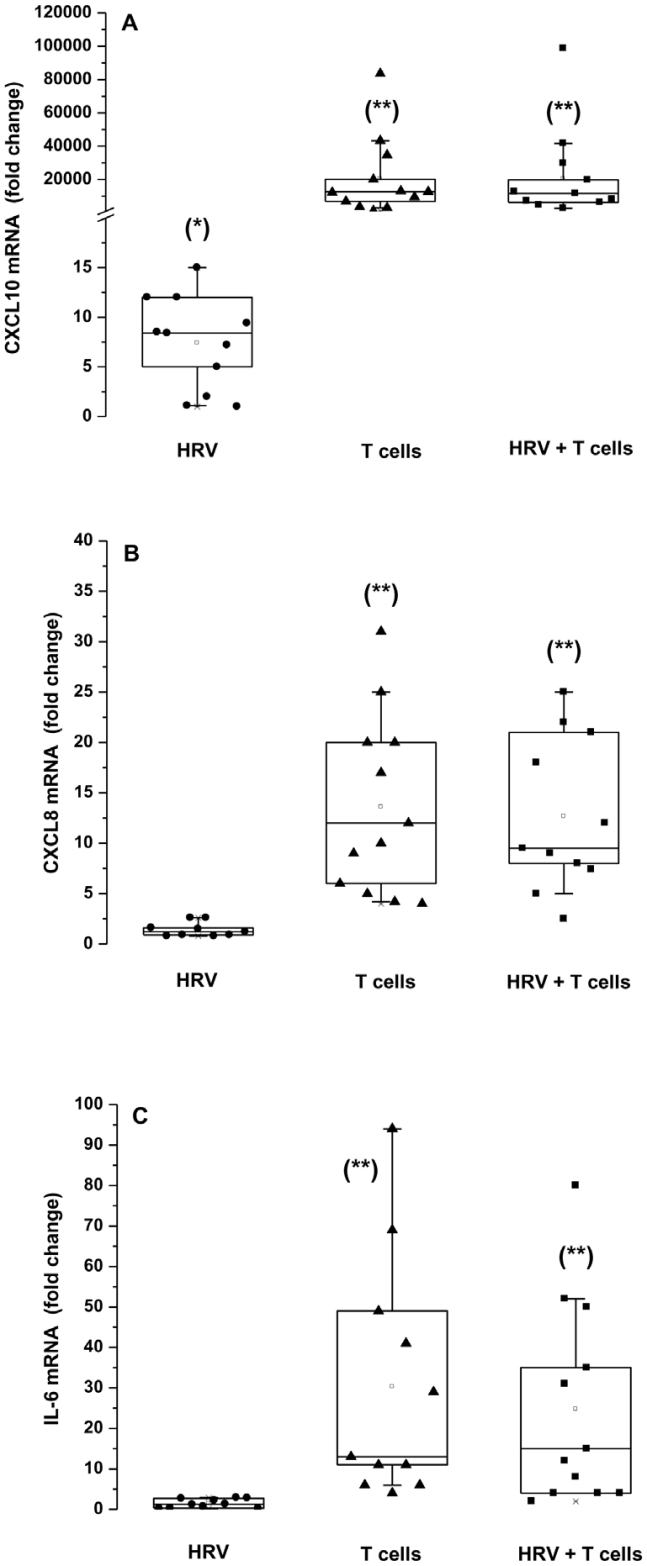
Epithelial CXCL10 mRNA is induced by HRV14 and T cells, CXCL8 and IL-6 mRNA are induced only by T cells. HNEC were exposed apically to HRV14 alone (HRV), or co-cultured with activated T cells alone (T cells), or exposed to HRV14 washed thoroughly then co-cultured with activated T cells (HRV+T cells). The expression of CXCL10 mRNA (A), CXCL8 mRNA (B) and IL-6 mRNA (C) was determined by real-time PCR. Data represent the fold increase relative to non-infected cells cultured in the absence of T cells. Shown are the boxplots with the 25^th^, 50^th^ (median), 75^th^ percentiles, the mean (open square), the maximum and the minimum values. *p<0.05 compared with control non-infected cells. **p<0.05 compared with HRV-infected cells.

HRV14 significantly increased mRNA expression of CXCL10, but did not affect that of CXCL8 and IL-6 in epithelial cells (Fig. 2A–B–C). Co-culture of activated T cells with HNEC boosted chemokines and cytokine production. In particular, epithelial CXCL10 mRNA expression increased considerably. Unstimulated T cells had no effect (data not shown).

**Figure 3:**
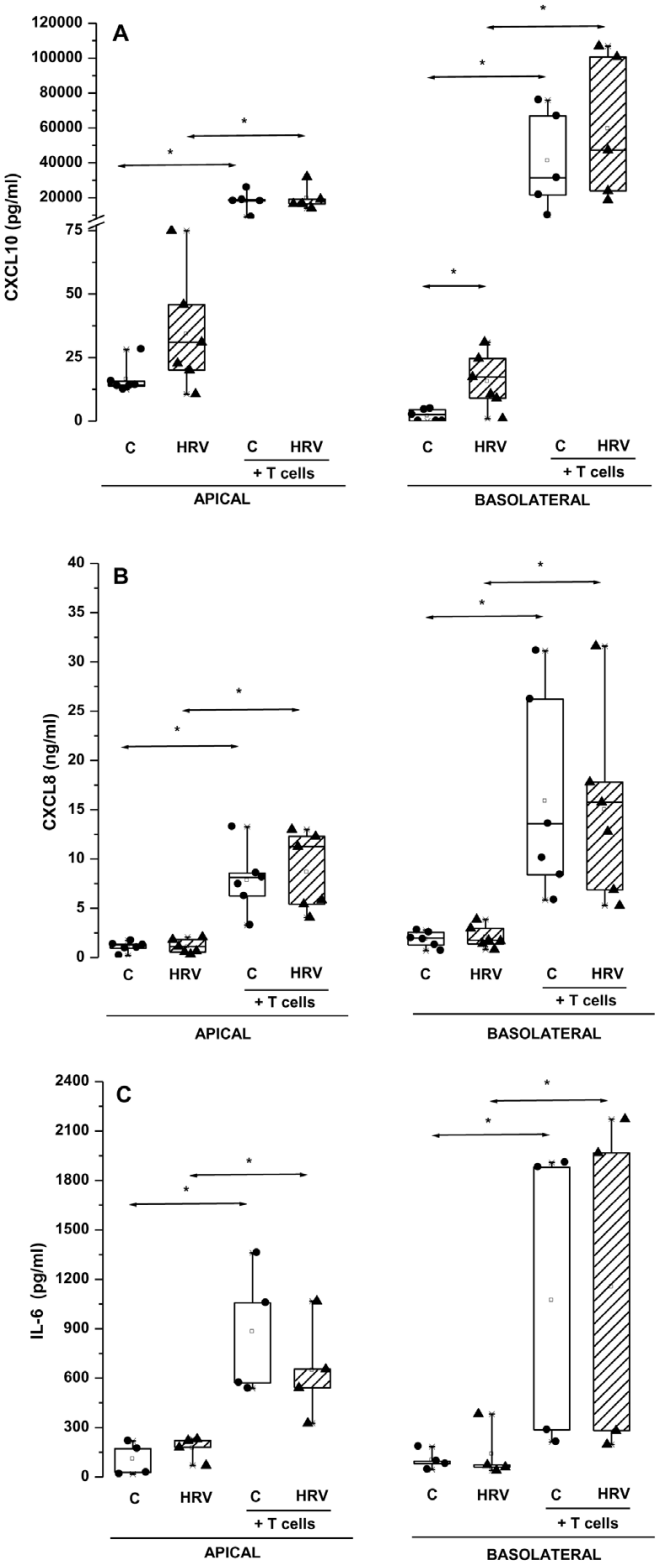
Both HRV14 and T cells enhance CXCL10, but only T cells enhance CXCL8 and IL-6 protein secretion by HNEC. HNEC were either not infected (C) or exposed apically to HRV14 alone (HRV), and co-cultured or not with activated T cells (T cells). The production of CXCL10 (A), CXCL8 (B) and IL-6 (C) was measured in apical and basolateral culture supernatants. Shown are the boxplots with the 25^th^, 50^th^ (median), 75^th^ percentiles, the mean (open square), the maximum and the minimum values. *p<0.05. In co-cultures experiments, the amounts of cytokines/chemokines released by epithelial cells in the basolateral compartments were determined by subtracting the amounts of cytokines/chemokines measured in the culture media of activated T cells cultures alone (undetectable for CXCL10, 0.034 ng/ml for CXCL8, 10.6 pg/ml for IL-6, median).

CXCL10, CXCL8 and IL-6 proteins were detected in both the apical and basolateral compartments (Fig. 3A–B–C). HRV14 induced a significant increase in basolateral secretion of CXCL10 (p = 0.03) without affecting that of CXCL8 and IL-6. Consistent with mRNA data, activated T cells co-cultured with HNEC greatly enhanced the apical and basolateral secretion of CXCL10, CXCL8, and IL-6.

The stimulating effects of HRV14 and activated T cells were neither additive nor synergistic.

### Anti-IFNγ and TNF soluble receptor p75 strongly inhibit CXCL10 and partially attenuate NOS2 mRNA expression induced in HNEC by activated T cells

**Figure 4:**
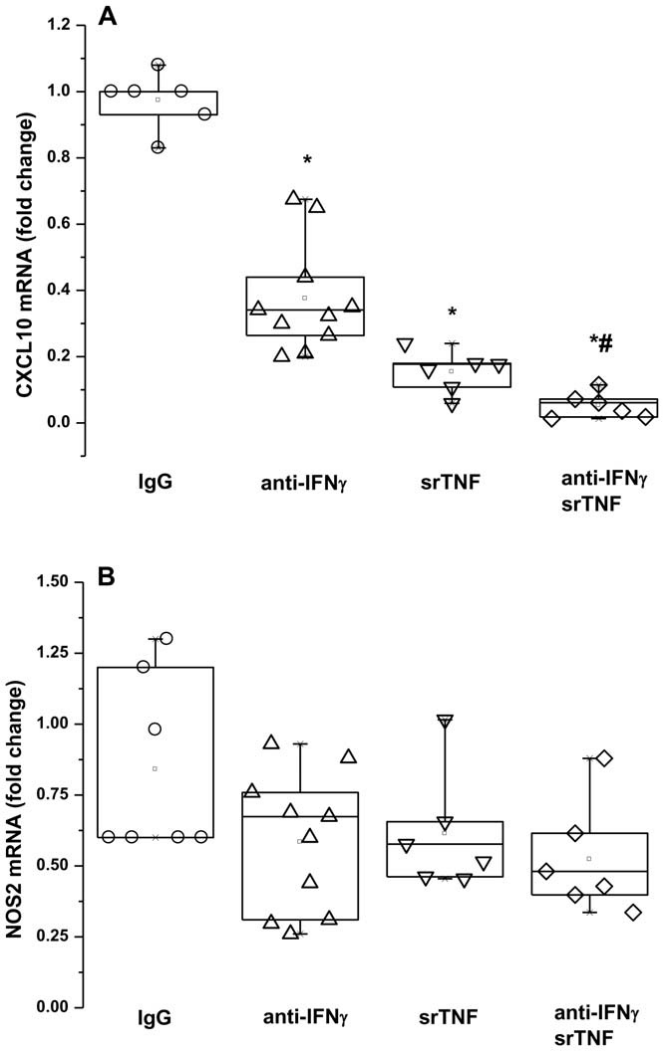
Anti-IFNγ and TNF soluble receptor p75 strongly reduce epithelial CXCL10 mRNA expression but only partially attenuate epithelial NOS2 expression induced by T cells. HNEC were co-cultured with activated T cells in the absence or presence of anti-IFNγ, TNF soluble receptor (srTNF) or an isotype-matched antibody (IgG). CXCL10 (A) and NOS2 (B) mRNA were measured by real-time PCR. Data are presented as fold change relative to those obtained in the absence of neutralizing agents. Shown are the boxplots with the 25^th^, 50^th^ (median), 75^th^ percentiles, the mean (open square), the maximum and the minimum values. *p<0.05 compared with isotype matched IgG treated cultures. #p<0.05 compared with anti-IFNγ and srTNF treated cultures.

Since CXCL10 gene promoter contains GAS (Gamma Activating Sequence) and ISRE (Interferon Regulatory Factor response Element) that are important for IFNγ-mediated gene expression, and NFκB (Nuclear Factor kappa B) that is important for TNF-induced gene expression [26], we asked whether the stimulating effects of activated T cells were mediated by IFNγ and/or TNF secreted by T cells in the media. We thus examined the effects of activated T cells in co-culture with HNEC in the presence of neutralizing anti-IFNγ and TNF soluble receptor p75. The T cells induced increase in epithelial CXCL10 mRNA expression was significantly reduced by 70±2% by neutralizing IFNγ with a specific antibody, and by 85±3% by neutralizing TNF with its soluble receptor p75. Neutralizing both IFNγ and TNF resulted in 95±2% reduction (Fig. 4A).

The T cells induced increase in epithelial NOS2 mRNA expression was reduced by 42±8% by anti-IFNγ, and by 47±3% by TNF soluble receptor p75 (Fig. 4B). The effects of anti-IFNγ and TNF soluble receptor were not additive or synergistic.

### Epithelial CXCL10 mRNA expression level induced by T cell clones strongly correlate with the secreted amounts of IFNγ and TNF

**Figure 5:**
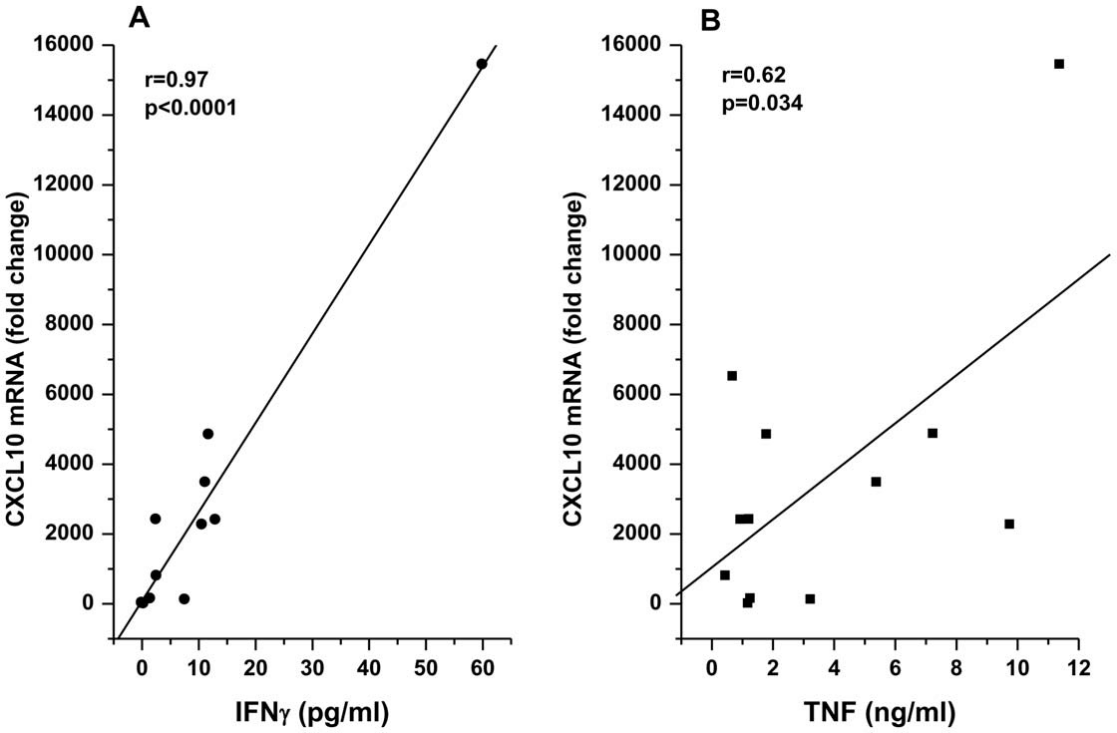
Epithelial CXCL10 mRNA induction is proportional to the amounts of IFNγ and TNF produced by T cell clones. HNEC were co-cultured with T cell clones obtained from PBMC of a normal individual. CXCL10 mRNA induction is expressed as a function of IFNγ (A) and TNF (B) secreted by T cell clones in the culture medium. Epithelial CXCL10 mRNAs are measured by real-time PCR and expressed as fold increase relative to those measured in epithelial cells cultured without T cells. IFNγ and TNF were measured by TenPlex bead immunoassay and ELISA.

We extended these findings by examining the effects of T cell clones that secreted different amounts of IFNγ and TNF, in co-cultures with HNEC. In concordance with data from neutralizing experiments, the degree by which T cells clones stimulated CXCL10 expression was proportional to their potency to produce IFNγ or TNF (Fig. 5A,B). When correlating epithelial CXCL10 mRNA levels with IFNγ protein secreted by T cell clones, a significant linear relationship could be detected (r = 0.97, p = 0.0001). We also observed a linear correlation between epithelial CXCL10 and T cell derived TNF, but the correlation was statistically less significant (r = 0.62, p = 0.034). Unstimulated T cell clones were ineffective (data not shown).

### Epithelial CXCL10 protein secreted into the basolateral compartment increases with increasing numbers of T cells and amounts of IFNγ and TNF, attaining a plateau at high concentrations

**Figure 6:**
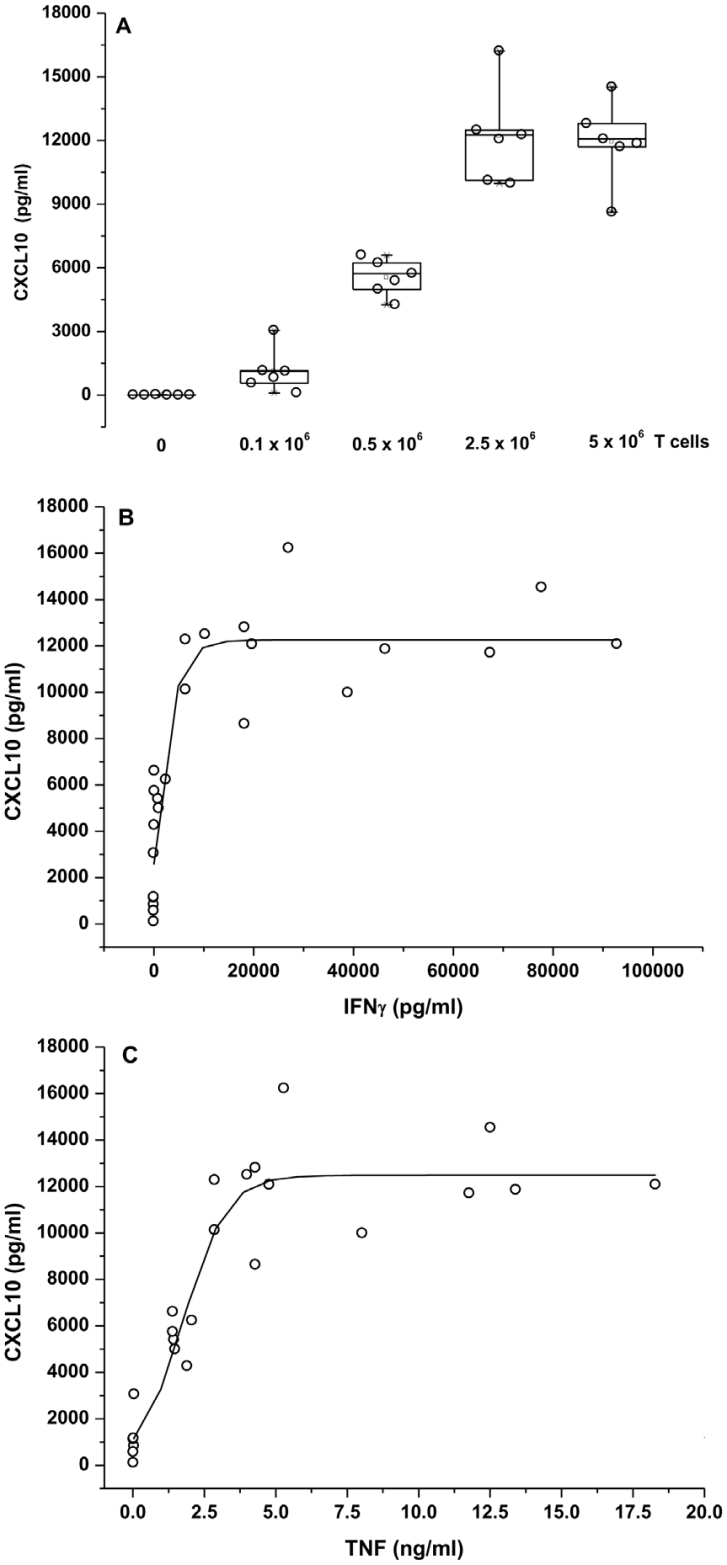
Epithelial CXCL10 protein secreted into the basolateral compartment increases with increasing T cells numbers and increasing IFNγ and TNF amounts, reaching a plateau at high concentrations. A constant number of HNEC (0.5×10^6^ cells) was co-cultured with increasing numbers of activated T cells (0.1, 0.5, 2.5, 5×10^6^ cells). CXCL10 protein amounts secreted by HNEC into the basolateral media are expressed as a function of the numbers of T cells in co-cultures (A) and the amounts of IFNγ (B) and TNF (C) secreted by increasing numbers of T cells.

Increasing numbers of activated T cells (0.1, 0.5, 2.5 and 5×10^6^) to a constant number of HNEC (0.5×10^6^) in co-cultures resulted in increased basolateral secretion of epithelial CXCL10 with a maximum at 2.5×10^6^ T cells (Fig. 6A). When correlating the amount of CXCL10 secreted by HNEC with the amounts of IFNγ and TNF secreted by increasing numbers of T cells, a saturation curve was obtained with a plateau at IFNγ level>10 ng/ml (Fig. 6B) and TNF concentration>5 ng/ml (Fig. 6C).

### Recombinant IFNγ and TNF induce epithelial CXCL10 mRNA expression, but at a level much lower than that induced by activated T cells

As neutralizing IFNγ and TNF largely abrogated T cells-induced epithelial CXCL10 expression, we examined if basolateral treatment of HNEC with a mixture of recombinant IFNγ plus TNF (50 ng/ml each) stimulated epithelial CXCL10 expression. A 48 h exposure of HNEC to IFNγ plus TNF resulted in 11+1.5 fold increase in CXCL10 mRNA level, much less than that observed in HNEC cultured in the presence of activated T cells.

### Activated T cells inhibit virus replication in HNEC partially through the nitric oxide pathway

**Figure 7:**
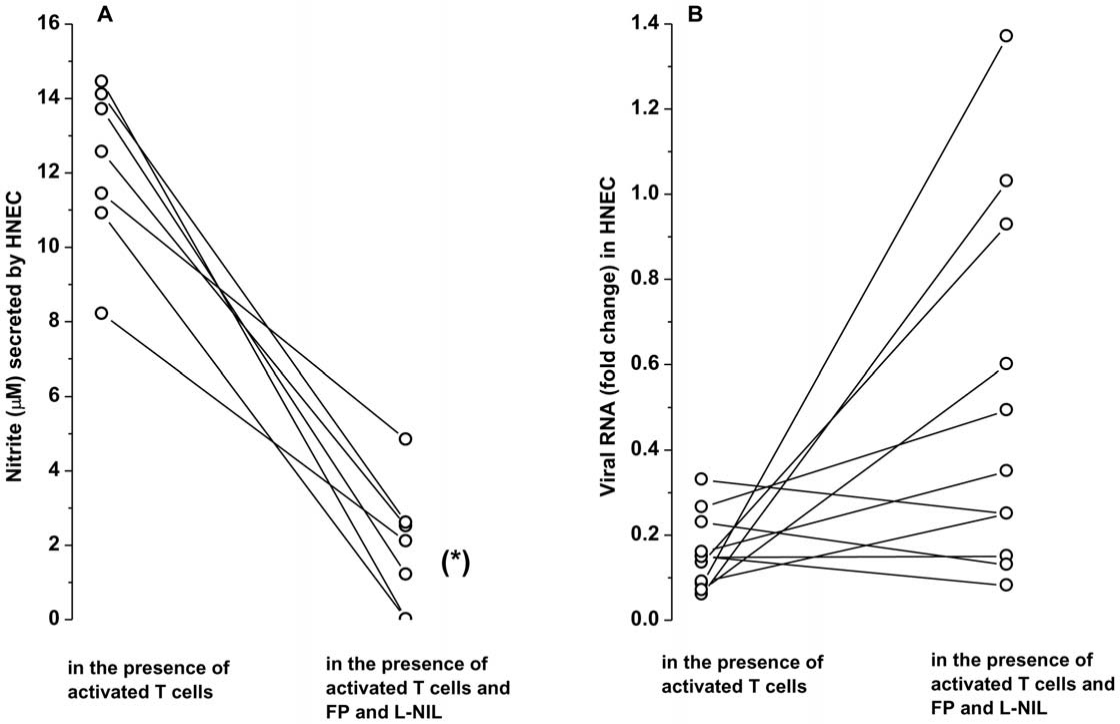
Activated T cells inhibit virus replication in infected HNEC partially through the NO pathway. HNEC were exposed apically to HRV14, washed thoroughly then co-cultured with activated T cells in the presence or absence of FP and L-NIL, inhibitors of NOS2 mRNA and activity, respectively. (A) NO secretion in the basolateral compartment and (B) viral RNA levels. NO is expressed as nitrite, the stable oxidation product of NO (µM). Viral RNAs was measured by real-time PCR and expressed as fold change relative to those observed in infected HNEC cultured in the absence of activated T cells. *p<0.05

NO inhibits viral replication [14]. Since activated T cells significantly induced NOS2 in HNEC, we asked whether this would impact HRV14 replication in infected HNEC. In the presence of activated T cells, the level of viral RNA in infected HNEC was reduced by 80.4±0.03% compared to that observed in infected cells cultured without T cells (p<0.05). To assess the role of NO in viral replication, we used FP and L-NIL that inhibits NOS2 mRNA expression and activity, respectively. Addition of FP and L-NIL in co-cultures resulted in 55±12% reduction in epithelial NOS2 mRNA, and 84±6% reduction in basolateral secretion of nitrite induced by T cells, respectively (Fig. 7A). Under these conditions, T cells were less effective in reducing viral replication since higher amounts of viral RNA were observed in infected HNEC, although the difference was not statistically significant (Fig. 7B).

## Discussion

In this study, we show that T lymphocytes promote the antiviral and inflammatory response of differentiated human airway epithelial cells to HRV, highlighting important cross-talks between epithelial and T cells.

After a short period (6 h) exposure to 5000 TCID_50_ of HRV14, the virus particles that enter differentiated HNEC are fully capable of replication. This might be due to the lack of type 1 IFNs and NO responses, two systems known to block the spread of virus infection. Indeed, we did not observe induction of IFNβ and NOS2 mRNA expression in HNEC, and did not detect IFNβ protein and NO in the extracellular media 40 h after HRV14 infection in the absence of activated T cells. Our inability to detect up-regulation of the genes important in antiviral defense cannot be ascribed to insufficient infection of HNEC since the viral dose used in our study did allow productive infection of HNEC. Of note, this viral dose is 5 times higher than that usually employed in experimental inoculation studies, reported to be effective in inducing significant cold symptoms[20], [24], [25]. Hence our observation in nasal epithelial cells differ from those previously reported for bronchial epithelial cells [27]. This may be related to the degree of infection, the cell's origin (upper or lower airways) and/or differentiation status. Of note, highly polarized cells like those used in our study were reported to be much less susceptible to virus infection [28]. In addition, no significant induction of the neutrophil attractant CXCL8 and the pro-inflammatory cytokine IL-6 was observed in our cells following HRV14 challenge, which is in accordance with data obtained with fully differentiated epithelial cells grown with an air-liquid interface [28], but in contrast with those obtained with undifferentiated tracheal/bronchial primary epithelial cells or cell lines grown as submerged monolayers [11]–[13]. We did observe a consistent and significant induction of mRNA and secreted protein of CXCL10, a potent chemo-attractant for T cells. This observation supports the current opinion that epithelial cells are the primary source of CXCL10 during HRV infection. It has been reported that CXCL10 production increased in airway epithelium after in vivo and in vitro HRV infection [6], [29]–[31], in airway secretions during symptomatic in vivo HRV infections, and in serum of asthmatic patients during virus-induced exacerbations [32]. Taken altogether, our results show that apart from CXCL10, HRV infection by itself did not induce significant antiviral and inflammatory responses in fully differentiated airway epithelial cells with intact tight junctions.

Interestingly, activated T cells alone promote a marked increase in CXCL10, IL-8, IL-6 and NOS2 expression in HNEC. The effect was particularly strong for CXCL10, thousands times greater than that induced by HRV14. Epithelial CXCL10 induction is mediated by T cells derived soluble factors because activated T cells were not in direct contact with HNEC in our co-cultures. Our data obtained with neutralizing antibodies, T cell clones secreting different IFNγ and TNF amounts, and increasing numbers of T cells, clearly indicate that IFNγ and TNF were the main inducers of epithelial CXCL10. Our observation that epithelial CXCL10 production attained a plateau at high IFNγ and TNF concentrations points to saturation of IFNγ/TNF receptors. However, recombinant IFNγ and TNF added as a single bolus at concentrations (50 ng/ml) equivalent or greater than those secreted by high numbers of T cells induced only a modest increase in epithelial CXCL10 mRNA expression. This might be due to the fact that exogenously added cytokines are short-lived in the culture media while in co-cultures, cytokines are continuously produced by activated T cells. Of note, exogenous TNF added alone had no effect (data not shown) implicating that in co-cultures the stimulating effect of TNF on epithelial CXCL10 might be mediated through INFγ induced by TNF. Taken altogether these data highlight the importance of continuous interactions between immune and epithelial cells resulting in uninterrupted production of cytokines to maintain transduction signals for host responses. The observed additive effects of IFNγ and TNF on CXCL10 suggest that more than one signaling pathway was involved, similar to signaling in human monocytes [33] and airway smooth muscle cells [34]. By contrast, the effects of IFNγ and TNF on NOS2 are not additive, suggesting that they might act on similar transduction pathways. Furthermore, IFNγ and TNF were only partially effective in inducing epithelial NOS2, indicating that other soluble factors were required.

A recent report has revealed active interactions between human bronchial epithelial cells, monocytes and HRV16 resulting in a synergistic induction of CXCL10 and CCL3 in bronchial epithelial cells. In the latter work, epithelial cells were submerged undifferentiated cultures, and in direct contact with HRV16 and monocytes [31]. As such, this experimental system is most likely representative of severe in vivo infection conditions with immune cells infiltrating the mucosa. Our experimental model of polarized airway epithelial cells cultured on semi-permeable membrane and separated from activated T cells is more appropriate to study early interactions through soluble factors when the epithelium is still intact after a mild HRV infection.

Interestingly, activated T cells markedly reduced virus replication in HNEC, may be through IFNs and/or NO known to activate different genes leading to apoptosis of infected cells, thereby limiting viral replication and release [3], [35]. A role for type 1 IFNs can be ruled out as we detected no increase in IFNβ gene expression in infected HNEC co-incubated with T cells (data not shown). In contrast, NO is involved in viral clearance, since in most cases when NO production is inhibited HRV14 RNA levels are higher in infected HNEC. This is in keeping with clinical observations reporting that patients with high levels of exhaled NO clear virus more rapidly and have lower symptom scores [14].

In conclusion, except for CXCL10, highly differentiated human airway epithelial cells are quite unresponsive to low level of HRV infection in terms of antiviral and inflammatory responses, which become largely significant in the presence of activated T cells. It is expectable that a mild viral infection of the mucosal epithelium in vivo promotes further airway inflammation by triggering epithelial CXCL10 production, allowing accumulation of activated T lymphocytes which in turn enhance epithelial chemokines and cytokines production. On the other hand, activated T cells recruited to the site of infection help epithelial cells to clear viruses through production of IFNγ and TNF, and induction of epithelial NOS2 expression. Consequently, epithelial cells are important effector cells in immune response and activated T lymphocytes are a major player in HRV-induced airway inflammation by linking innate and adaptive immunity.
